# Academic Burnout of Polish Students: A Latent Profile Analysis

**DOI:** 10.3390/ijerph20064828

**Published:** 2023-03-09

**Authors:** Teresa Chirkowska-Smolak, Magdalena Piorunek, Tomasz Górecki, Żaneta Garbacik, Violetta Drabik-Podgórna, Anna Kławsiuć-Zduńczyk

**Affiliations:** 1 Faculty of Psychology and Cognitive Science, Adam Mickiewicz University, 60-568 Poznań, Poland; 2 Faculty of Educational Studies, Adam Mickiewicz University, 60-568 Poznań, Poland; 3 Faculty of Mathematics and Computer Science, Adam Mickiewicz University, 61-614 Poznań, Poland; 4 Institute of Pedagogy, University of Wrocław, 50-527 Wrocław, Poland; 5 Faculty of Philosophy and Social Sciences, Nicolaus Copernicus University, 87-100 Toruń, Poland

**Keywords:** professional burnout, academic burnout, education, latent profile analysis

## Abstract

According to Maslach and Leiter, burnout syndrome consists of three elements: exhaustion, cynicism, and a sense of ineffectiveness experienced by individuals in the work environment. However, burnout does not only apply to professional activity but can also be experienced by students pursuing higher education. This is important because the consequences of student burnout can be related to students’ mental and physical health. Until recently, the dominant diagnostic trend in the studies of burnout syndrome was based on a variable-focused approach. This approach focuses primarily on identifying subgroups in the population and presents different configurations of the various dimensions of burnout. However, there is emerging research using a person-centered approach and including the analysis of latent profiles to study professional and student burnout. This approach allows us to isolate subgroups of individuals in the study sample who have a similar burnout pattern. It focuses on the differences between individuals, which helps us to look at the phenomenon of professional burnout from a different perspective and shows the individuality of its experience. Our research aimed at identifying latent profiles, was conducted on a sample of 1519 Polish students, and partly confirms reports from other countries. We identified four profiles: low burnout, moderate below-average burnout, moderate above-average burnout, and very high burnout groups.

## 1. Introduction

The increasing workload placed on employees in today’s labor market, the growing complexity and number of tasks undertaken, and time pressures to perform such tasks contribute to raising the risk of burnout among employees. However, this is not a phenomenon limited only to the professional environment. With the increased interest in student health and well-being [1] (p. 249), it has also become clear that stress and the threat of burnout affect not only professionals but also university students [2,3,4], representing significant groups of people preparing to enter the labor market. As UNESCO data show, the number of students worldwide has more than doubled from 2000 to 2020 [5]. This means that professional burnout may be a problem for a significant part of the population, especially as the number of people entering higher education is assuming an upward trend. Students often have to cope with high cognitive demands and time pressure, which increases the risk of the onset of burnout [2,6]. In addition to the stressors of studying and examinations, they also have to face the transition to university, entering a new peer group, taking on responsibility in the face of new duties, financial difficulties, and disconnection from the familiar environment, home, and family [7].

The consequences of student burnout also deserve attention. As the research shows, burnout can influence the decision to drop out of studies, which can generate personal and social costs (including dropout) [8,9,10], especially since it is also associated with poor academic performance [11]. In addition, it influences students’ mental and physical health [12] and even can be connected with the emergence of suicidal ideation [13,14]. Burnout is also indicated as one of the variables (along with commitment and social support) affecting drug use among students [15]. As for social support, its protective role against burnout [16] may be demonstrated, as burnout affects relationships with colleagues and teachers [12].

Following Christina Maslach, Wilmar Schaufeli, and Michael Leiter, we can assume that burnout is a gradual process during which the mismatch between the needs of the individual and the demands of the job increases [17]. In the International Classification of Diseases (11th revision), burnout is defined as “a syndrome conceptualized as resulting from chronic workplace stress that has not been successfully managed. It is characterized by three dimensions: (1) feelings of energy depletion or exhaustion; (2) increased mental distance from one’s job, or feelings of negativism or cynicism related to one’s job; and (3) a sense of ineffectiveness and a lack of accomplishment” [18]. Referring to the multidimensional model of burnout, Schaufeli, Salanova, Gonzalez-Roma, and Bakker [19] indicated that it is a phenomenon that involves in the case of students “feeling exhausted because of study demands, having a cynical and detached attitude towards one’s study, and feeling incompetent as a student” (p. 73). Analogous to how the phenomenon manifests in employees, among burned-out students, we observe such symptoms as a sense of excessive work overload [20,21], loss of enthusiasm for the subject studied and an impression that what one is learning does not make much sense [21], anxiety and class absences, lack of activity in class (decreased motivation), and deteriorating relationships with other students, academic staff, and family [22].

Burnout, although it has a confirmed three-factor structure (exhaustion, cynicism, and inefficiency) [23,24,25], does not develop in everyone in the same way and has an individual character in each case. It poses quite a challenge to adequately show the complexity of this phenomenon. Until recently, mainly variable-centered approaches were used, e.g., [2,7,25,26,27,28,29], with the research focusing primarily on identifying subgroups in the population. Associations of burnout in each dimension were analyzed separately with other variables, e.g., with its personality correlates or differences in the intensity of the phenomenon over time.

Currently, it has been increasingly acknowledged that the adequate investigation and testing of the complex phenomenon of burnout should go beyond conventional analytical schemes [30]. As researchers are being encouraged to think creatively about how these strategies can be applied to raise new questions, there has been increasingly more research on person-centered strategies in the study of burnout. Among these, an increasing number of such studies apply the LPA method. Several ones can also be identified in the area of student burnout, e.g., [3,31,32,33,34]. In the next section, we describe more extensively the examples of research conducted with LPA. However, first, we outline the difference between these types of research and explain the benefits of supplementing the existing knowledge with a person-centered approach.

### Person-Centered Approach and Latent Profile Analysis (LPA) in Burnout Research

The person-centered approach is clearly applicable to individual diagnosis by using the norms in the test manual. This is because we assume that the configuration of scores on individual scales and on specific subscales within them may be unique to each individual. In quantitative research, we traditionally use a variable-centered approach, relying on the assumption that all individuals in the sample are from a single population and that a single set of mean parameters can be estimated. The person-centered approach, used to quantitatively study phenomena in larger populations, relaxes this assumption. Researchers take into account the possibility of having different subpopulations in the sample characterized by varying sets of parameters [35]. By isolating subgroups of individuals who are characterized by a similar pattern within a population, we focus on the differences between individuals, which allows us to examine the phenomenon of burnout from a different perspective and shows the uniqueness of its experience.

Furthermore, by using the person-centered approach in the analysis of burnout, we recognize its complexity, taking into account the system of variables that may be interrelated, as, for example, different components of the multidimensional phenomenon of burnout interact with one another in complex ways, which we analyze in combination rather than in isolation. In this way, we can identify complex interactions between variables that are difficult to detect or interpret using a traditional analytical framework [36]. It seems that incorporating this still-new approach will allow us to look at the burnout phenomenon from a different perspective.

Leiter and Maslach [37] demonstrated that an innovative, person-centered approach to assessing experiences in the workplace or education is possible, going beyond the description of the phenomenon in the three correlated dimensions. Such an approach is known as the latent profile analysis (LPA). This person-centered statistical method is still relatively rarely used in the diagnosis of burnout. The growing interest in the use of this method to measure burnout prompted us to use it in the research presented here to demonstrate its utility in psychological measurement. We also believe that such a diagnostic perspective could be a starting point for developing preventive measures or measures related to the reduction of possible negative effects of burnout in higher education.

Relying on Christina Maslach’s three-dimensional model evoked above provides an opportunity to consider a more complex structure of the results, which goes beyond the possibility of calculating a single common score for burnout in general. After all, burnout includes different aspects, and the scores in individual dimensions do not necessarily coincide; they do not go “hand in hand”. The three dimensions of burnout are not even strongly correlated (especially the third dimension, the sense of efficacy, which is therefore controversial [38,39]), so the results cannot be simplified by reducing them to a single total score.

In practice, this means that if a person scores very high on the exhaustion scale and average on the cynicism and inefficacy scale, their averaged score would be the same as that of a person scoring very high on the cynicism scale and average in the other two dimensions. In the former case, we would be dealing with an exhausted person, and in the latter, with a cynical person, while their averaged scores would be similar. Therefore, calculating an overall burnout score would not allow us to see these individual patterns. In addition, a negative score on only one subscale could be a warning sign of developing a burnout, and averaging the score on the three subscales would make such an observation difficult, if not impossible, as the overall burnout score would be at a moderate level.

Hence, the authors of measurement tools recommend presenting the results on separate subscales [40,41]. Thus, when measuring professional burnout as a multidimensional construct, the results on each subscale are analyzed separately—the presence of burnout risk is inferred on the basis of specific combinations of results obtained on all subscales simultaneously (high-high-low).

Thus, the results of studies presented over the last few decades have shown the level of burnout (in different groups) in its three dimensions but have not given us a more accurate picture of the phenomenon with individual patterns in a specific population. A precise outline of such an account based on the results of a diagnosis is extremely important to be able to develop effective organizational intervention programs.

To look more closely at the unique calculations of this phenomenon in a population, various approaches can be used, e.g., dividing scores on individual subscales into high and low scores using a median. However, this may lead to oversimplifications, as scores slightly above or slightly below the median are assigned to two extreme groups. An analysis that identifies specific subgroups in the sample might be a better method. Statistical methods, which allow a customized approach to analyzing burnout and involve looking for patterns specific to a population, are based on the concept of data mining. The analyst looks for structures in the data without specific a priori assumptions as to how many clusters there are in the sample; this number is automatically determined by the applied algorithm. A valuable tool for classifying objects into meaningful groups can involve the analysis of latent profiles (LPA), which is a model-based variant of traditional nonhierarchical cluster analysis procedures, such as the K-means method. This approach facilitates distinguishing subgroups of people similarly experiencing burnout, however small in size, which will best reflect the data [42].

A certain regularity can be noticed in the profiles distinguished on this basis. Christina Maslach and Michael Leiter referred to multiple distinct patterns along the burnout–engagement continuum; they pointed out the two standard endpoint patterns of burnout (high in exhaustion and cynicism and low in professional efficacy) and engagement (low in exhaustion and cynicism and high in professional efficacy). They also identified profiles of people who only experienced one of the dimensions rather than all of them [40]. Anne Mäkikangas and Ulla Kinnunen, in their systematic review of burnout research [30], observed that researchers making typologies of burnout individuals most often distinguished between synchronous profiles, with scores in all dimensions being either high or all low, or profiles that were characterized by de-synchrony between burnout symptoms, with high scores in two dimensions or only in one dimension, mainly on the exhaustion scale.

Studies by Michael Leiter and Christina Maslach [37], using latent profile analysis, distinguished profiles that may deviate from the typical pattern emerging when burnout dimensions are assumed to be correlated, i.e., profiles with high scores in only one dimension. The results of this study indicate the presence of five employee profiles, including two profiles with high scores in all dimensions and low scores in all dimensions (scores in the third dimension were recoded by the researchers, with a high score indicating inefficacy) and three profiles with high scores on one scale only. These were the profiles of the following individuals: (1) burned out, scoring high in all dimensions; (2) engaged, with low scores in all dimensions; (3) exhausted, having high scores only in the exhaustion dimension and moderate scores in the other two; (4) unengaged, with high scores only in the cynicism dimension and moderate scores in the other dimensions; and (5) ineffective, scoring high on the reduced efficacy scale and moderate on the other two. In the two samples of Canadian healthcare workers analyzed by Leiter and Maslach (N1 = 1766, N2 = 1166), participants in the one-high-dimension profiles accounted for half of the respondents (49%).

The LPA has also been used to analyze academic burnout. A study by Igor Portoghese, Michael Leiter, Christina Maslach, Maura Galetta, and Fabio Porru et al. [3] of Italian university students showed a similar pattern of correlations, with two profiles of individuals with related scores in three dimensions (i.e., burned out and engaged) and one profile of overextended individuals characterized by high scores in one dimension of exhaustion and moderate scores in the other dimensions of cynicism and efficacy in their academic work. Students assigned to this atypical profile accounted for as much as 51% of the sample. However, the authors did not distinguish between the two profiles with high scores on only one scale as revealed in Leiter and Maslach’s study, i.e., cynical and ineffective.

Latent profiles were also the subject of a study conducted by Kati Vinter [34] on the profiles and coping patterns of adolescents in Estonia. The study aimed to identify latent profiles of Estonian high school students who revealed different levels of academic burnout over the course of the school year and, most importantly, to examine differences between the identified profiles in the context of academic buoyancy and cognitive emotion regulation. Eventually, two profiles were identified, which were called “above-average burnout” and “below-average burnout”. The study found that the number of students who experience above-average burnout throughout the school year was unexpectedly high (40%) compared to peers in the other group. Students in the “above-average burnout” group reported significantly lower levels of buoyancy. Compared to the “below-average” group, in all measurement periods, they relatively less frequently used cognitive adaptive emotion-regulation strategies (especially the positive reappraisal strategy) and more frequently chose non-adaptive strategies (especially blaming, ruminating, and catastrophizing).

The work of Asian researchers from China or South Korea may also be an example of research conducted in groups of students. Xiaozhou Zhang, Robert Klassen, and Yun Wang [43] studied high school students in China. They identified four burnout profiles: two with high/low scores in three burnout dimensions (distressed group and well-functioning group profiles, respectively) and additionally two profiles with mixed scores. These were as follows: the persevering group, with high scores on the exhaustion and cynicism scales but low scores on the academic inefficacy scale, and the reverse profile of the laissez-faire group, with low exhaustion and cynicism but higher scores on the inefficacy scale. Min Young Lee, Mi Kuong Lee, Min Joo Lee, and Sang Min Lee [44] considered, in addition to exhaustion, cynicism, and inefficacy, two additional factors as important in Korean cultural background students. These new factors include anxiety and resentment (antipathy), which reflect Korean students’ negative attitudes toward learning and school. The clusters distinguished by measurement using the KABI five-dimensional burnout scale show that the authors identified two groups with high and low scores on all five scales (distressed group and well-functioning group profiles, respectively). In addition, they distinguished two more profiles of students: the laissez-faire group, with low scores on the exhaustion, inefficacy, and anxiety scales and higher scores on the cynicism and antipathy scales, and the struggling group, with high scores on the exhaustion, inefficacy, and anxiety scales and low scores on the cynicism and antipathy scales.

We would also like to cite the results of another study that used the LPA method, but the analyses were related to a different theoretical framework. It was mentioned above that burnout and engagement can be understood in different ways. Burnout was originally defined as the state in opposition to positive engagement [45] and regarded as the extremes of the same three dimensions of energy, identification with work, and effectiveness. Engagement, as suggested by Maslach and Leiter, [46] is a positive work experience given the absence of burnout symptoms and thus can be measured by the same tool (MBI). It should be noted, however, that in recent years, there has been an ongoing debate among researchers as to whether they are the exact opposites and whether the absence of negative symptoms should be equated with a positive state of mind. It can be inferred that the relationship between burnout and engagement is more complicated than it has been stipulated at the conceptual level. Some researchers also raise doubts about the factorial structure or the existence of the dimensions located between burnout and engagement [47]. Although Maslach and Leiter [46] proposed an approach according to which burnout and engagement are opposite poles of the same dimension, many authors treat engagement, following Schaufeli et al. [47], as a separate phenomenon. For the purposes of this study, we adopted the approach with burnout and engagement as separate constructs and interpreted the low scores on the questionnaire as indicating students’ lack of burnout.

For example, such a study was carried out in Finland by Katariina Salmela-Aro and Sanna Read [31], in which the authors looked for profiles that would simultaneously include burnout and engagement as two independent phenomena (albeit negatively correlated with each other). A large and representative sample of 12,394 students from universities and technical colleges/polytechnics was examined. As a result, four profiles were identified, each representing a different combination of engagement in learning and burnout: engaged (44%), engaged-exhausted (30%), inefficacious (19%), and burned out (7%). Two of these profiles were standard, with similar scores in all three dimensions of burnout and the three dimensions of engagement: engaged, with high scores on the engagement scales and at the same time low scores on the burnout scales, and burned out, with high scores on the burnout scales and low scores on the engagement scales. Two additional profiles highlighted by the authors are asynchronous: engaged but exhausted, with high scores on the engagement scales and, at the same time, a high score on the burnout scale, and burned out, with only one high score, indicating heightened experience of inadequacy as a student. The engaged group consisted of students in the early stages of studying (undergraduate studies), while the ineffective and burned-out group involved students who studied the longest. In other words, as the number of years of studying increases, the burnout increases, and engagement in learning decreases, and this relationship was present both at universities and polytechnics. Cynicism and inefficacy increase gradually with the number of years of studying. Given that some students find it difficult to complete their higher education, thereby prolonging their studies, they may be particularly vulnerable to burnout due to the increasing stress and doubt about the purpose of studying. Another important finding of this research involved the identification of a large group of students who were simultaneously exhausted and engaged (30%), providing insight into the dark side of engagement.

The results of these studies show that burnout is a complex phenomenon and can be a varied experience for many people. It is only by distinguishing specific profiles that a “tailor-made” intervention program can be developed. For example, in the case of Italian students, in addition to constructing a program to help students who are fully burned out, it is also necessary to focus on preventive intervention involving the development of strategies for managing workloads by universities.

Given the advantages of the person-centered strategies and the paucity of knowledge about burnout profiles among Polish students, we decided to try to include data from our home country in a review of research conducted in this relatively new trend. The main objective of our study was to identify burnout profiles in a group of Polish students using LPA. Based on Leiter and Maslach’s [37] study of burnout profiles in employees, we expected to be able to distinguish five profiles. The first include two standard profiles of high and low burnout, which reflect both ends of the burnout continuum, with either high or low scores on all three MBI scales. The next are three atypical, i.e., inconsistent, profiles with high scores on only one MBI scale, proposed by Leiter and Maslach and based on earlier longitudinal studies [40]: exhausted (overextended), with high scores on the exhaustion scale and lower scores on the other two; cynical (disengaged), with high scores only on the cynicism scale; and ineffective, with high scores only on the inefficacy scale (reversed scores for professional efficacy).

Thus, the hypothesis we put forward is that the MBI-SS scale would cluster into five profiles, reflecting a pattern of three correlated scales (two profiles of severe burnout and not burned out) and indicating the presence of atypical patterns of dependency, with high scores on only one scale (three profiles of exhausted, cynical, and ineffective).

**Hypothesis** **1.** *The two standard profiles will be the endpoints of the high level of burnout (all three scales show high scores) and the low level of burnout (all three scales show low scores)*.

**Hypothesis** **2.** *The three atypical profiles will show high scores on only one scale: high exhaustion only (overextended), high cynicism only (disengaged), and high inefficacy only (ineffective)*.

## 2. Materials and Methods

The aim of our study was to determine whether the three dimensions of burnout form individual profiles in those experiencing burnout or whether there is a generally negative phenomenon of burnout. We conducted our analyses in relation to academic burnout in students. For this purpose, we used the person-centered approach, i.e., LPA.

### 2.1. Participants

The study was conducted from May to July 2021 at four large, public universities in Poland (in important academic centers in the country, including Poznań, Toruń, Warsaw, and Wrocław). As many as 1519 people participated in the study (the number of people who sent questionnaires complete with answers to metric data questions). They were students of education and social sciences. Among them, there were such majors as pedagogy, special education, preschool and early childhood education, and psychology.

The sample was predominantly female, as women accounted for 93.55% of the respondents, which is due to the feminization of the studied subjects. The respondents studied in two different modes, i.e., full-time and extracurricular. Among those surveyed, 70.40% were full-time students. Despite the predominance of full-time students, a relatively large number of respondents (41.22%) indicated they had already taken up employment. This confirms the trend observed in Poland for young people to pursue additional employment while studying regardless of the mode of studies. The respondents studied in two different programs of studies, which are conducted in parallel in Poland. Some of them were in five-year master’s degree programs, while others pursued two-degree studies in the so-called Bologna system adopted in the EU. Two-degree studies consist of a three-year bachelor’s degree and a two-year master’s degree. Completion of a bachelor’s degree does not compel one to continue on to a second degree program, although in the case of students majoring in education, this is often the case. The sample included 48.99% of undergraduate students, 26.80% of graduate students, and 24.21% of students in a five-year degree program. Among the respondents, 38.52% were first-year students, i.e., those who have been in higher education for less than a year (given that the survey was conducted at the end of the academic year). A similar number of them (36.32% of those surveyed) were those in their final year; i.e., they were formally completing a given cycle of education in a few months after the study at the latest.

Information about the study being conducted and the possibility of voluntary participation in it was disseminated by means of messages addressed to students posted on the departmental websites, social media, and student mail. The information about the study indicated its objectives, how it was carried out, and the location of the diagnostic tools used and was prepared using MS Forms in MS Teams. The questionnaire was only available to those with active access to student mail, i.e., those with current student status.

It must be added that the present study was conducted in accordance with the recommendations of the local ethics committee at Adam Mickiewicz University. It included nonclinical surveys and applied noninvasive measures (self-ratings). Moreover, a cover letter guaranteeing confidentiality and explaining the purpose of the survey was attached. No treatment or false feedback was given, and no potentially harmful evaluation methods were used. Participation was completely voluntary, and participants were given an opportunity to resign at any time without negative consequences. Additionally, written online informed consent to participate in the survey was given by the respondents who clicked the “I accept” option.

### 2.2. Measures

To measure academic burnout, the Maslach Occupational Burnout Inventory–Student Scale (MBI-SS) was used [40]. Upon entering the study, the authors did not have a Polish adaptation of the MBI in the student version. The study was preceded by the development of a Polish-language version of the tool and confirmation of its psychometric value. First, the items were translated into Polish, and then, a reverse translation into English was made by a translator unfamiliar with the original version (blind translation). The reverse translation was compared with the original version. After ensuring that both versions were equivalent, we conducted a validation study. To validate the tool, we checked its factorial structure, verified the fit of the three-factor model (detailed results of the confirmatory factor analysis are presented later in the article), and calculated the reliability of the tool (its internal consistency). After conducting an exploratory factor analysis, we noticed that one item, MBI_6 (“I feel burned out from my studies”), definitely loaded higher on the CY factor, whereas in the original version, it is part of the EX factor. Excluding this item improved the fit of the three-factor model.

The Polish version used in this study consists of 15 items assigned to three subscales: exhaustion, associated with the demands of studying (EX, 4 items, e.g., “I feel worn out at the end of the day at university”); cynicism, denoting cynical attitudes and a lack of identification with one’s studies (CY, 5 items, e.g., “I doubt the significance of my studies”); and professional (academic) efficacy, i.e., the feeling that one is not a competent student (EF, 6 items reverse scored, e.g., “During class, I feel confident that I am effective at getting things done”). The scores for original professional efficacy scale (with the positively worded items) were reversed so that high scores reflected high inefficacy in order to be indicative of burnout.

Respondents provided answers on a 7-point Likert scale ranging from 0 (never) to 6 (every day). High scores on the exhaustion and cynicism scales suggest the occurrence of burnout in the students surveyed. In the case of academic efficacy, all items positively worded were reversed so that high scores reflected high inefficacy. It should also be added that an overall score for burnout was not to be calculated, e.g., as an average of all test items/items. The results of each subscale were analyzed separately, and individual profiles were created for each respondent.

The reliability of the subscales both in the original and in our research was good; in this study, Cronbach’s alpha was 0.84 for exhaustion, 0.90 for cynicism, and 0.81 for inefficacy.

The database was archived in the Open Science Framework (OSF) repository: https://osf.io/j9akt/ (accessed on 26 January 2022).

## 3. Statistical Analysis

All calculations and plots were prepared in the R version 4.1.1 package [48] using the lavaan library [49].

In the first step, we performed a confirmatory factor analysis (CFA) to confirm the three-factor data structure [50]. For the CFA, we used goodness-of-fit indices to evaluate the model. Comparative fit index (CFI) and Tucker—Lewis index (TLI) cutoff scores should be above 0.95. A root mean square error of approximation (RMSEA) value of less than 0.05 can be said to indicate a convergence fit of the analyzed data to the model. Standardized root mean square residual (SRMR) can be interpreted as an indicator of good model fit when it produces a value of less than 0.05.

In the next step, we performed latent profile analyses of the factors obtained from the CFA. We tried applying different models and finally decided to choose the model with alternating variances and covariances. (Based on [51,52], this model was retained as the best fit to the data given the low value of log likelihood, Akaike information criterion (AIC), Bayesian information criterion (BIC), sample size adjusted BIC (SABIC) value, and high entropy value.) Such a model allows variances and covariances to be freely estimated across profiles. Thus, it is the most complex model with the potential to facilitate an understanding of many aspects of the variables used to estimate the profiles and how they are related. The differences between the models were minor, so we decided to compare them, and we conducted a MANOVA analysis (multivariate analysis of variance). Finally, we decided to perform post hoc analyses (Tukey’s honest significant difference (HSD) test) to find statistical differences between profiles.

## 4. Results

**Figure 1:**
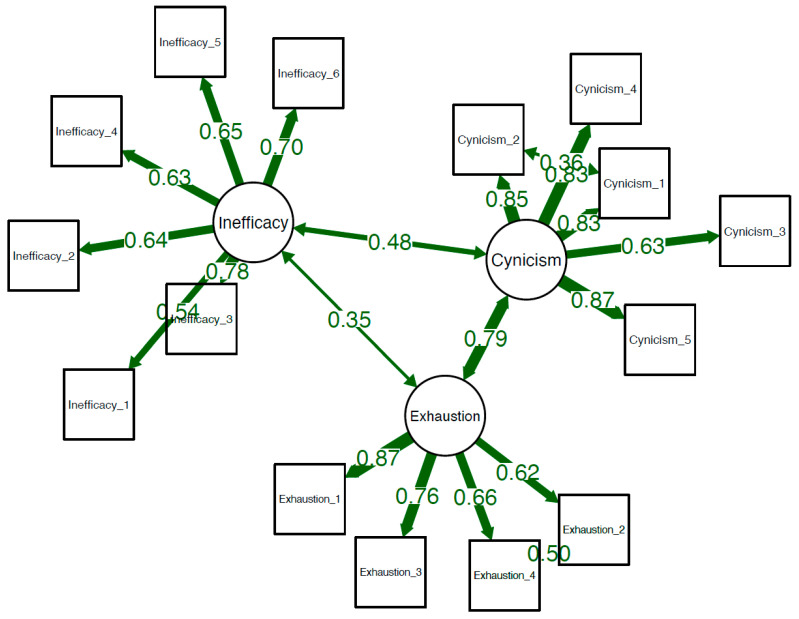
Graphical presentation of the confirmatory factor analysis model used.

For CFA, we obtained the CFI value of 0.990 and TLI value of 0.986, which indicate a very good fit and the internal validity of the model. The RMSEA is 0.033, and the SRMR is 0.028. In summary, we obtained very good values for all CFA fit indices. A graphical summary of the confirmatory factor analysis model used is shown in Figure 1.

**Figure 2:**
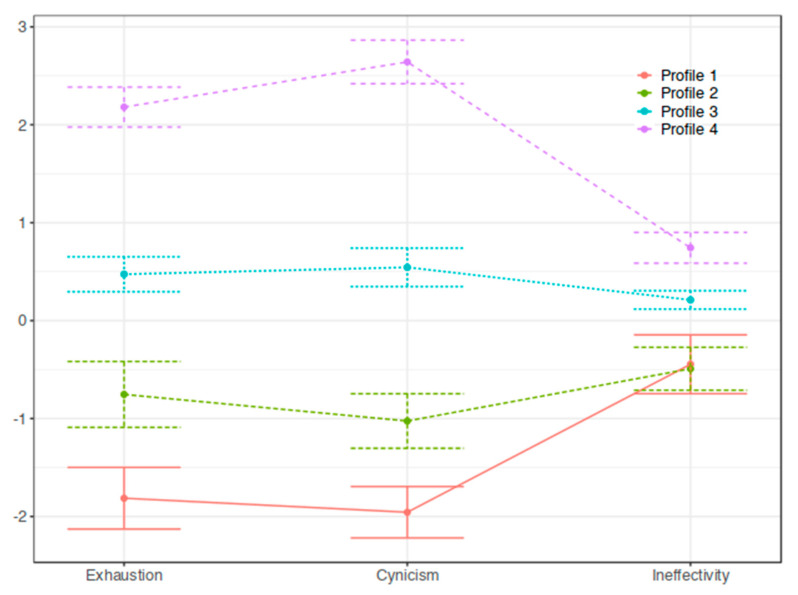
Line graph with 95% confidence intervals comparing profiles on indicator variables in z−score format.

From the above results, we were able to move on to the next step, in which we performed latent profile analyses of the factors obtained from the CFA. The proposed latent models for different numbers of profiles were assessed using the fit indices described below (Table 1). The differences between the models were minor, so we decided to apply the model with four profiles. To validate our decision, we conducted MANOVA analysis. In both cases (four and five profiles), all profiles were different (*p*-value = 0 in Pillai’s trace test). In the next step, therefore, we decided to perform post hoc analyses (HSD test) to see which profiles specifically differed. In the case of four profiles, all profiles were different in all features. On the other hand, in the case of five profiles, we obtained four disjointed profiles in the case of exhaustion and even three profiles in the case of inefficacy. Only for cynicism did we obtain five disjointed profiles The selected model is detailed in Table 2 and Figure 2. The means and standard deviations of variables used to create the profiles are presented for each profile.

As MBI authors suggest, the results can be interpreted as absolute values or by comparing them to the results obtained in a larger population. Unfortunately, when we analyzed the results of the present study, the current test manual (MBI Manual 4th Edition) did not contain data to interpret the results of the student version. Therefore, in interpreting the results as high or low, we followed the suggestions of Leiter and Maslach [37], who defined profiles by referring to standardized scores. These authors established the criteria for categorizing respondents by profile type using standardized values for the examined sample. For example, in the burnout profile proposed by Maslach and Leiter, the pattern is EX high, CY high, and PE (professional efficacy) low. To interpret the profiles, we also relied on the standardized results, but in our sample, we observed a profile of severely burned out with EX results that were very high (above two standard deviations), CY results that were even slightly higher than EX, and INEF results (as mentioned earlier, we reversed the obtained results on this scale) of a moderate level. We interpreted the results in the area of one standard deviation from the mean as moderate, from +/− 1 SD to 2 SD as high/low, and in the area of +/−2 SD to 3 SD from the mean as very high/very low.

Based on this, we distinguished four profiles, which are as follows (Figure 2): (1) “low burnout” (18%)—students belonging to this group scored low on the EX and CY scales (which, given the 95% confidence interval, can also be interpreted as very low) and moderate on the INEF scale; (2) “moderate below-average burnout” (22%)—obtaining, given confidence intervals, from low to moderate results on the EX and CY scales and moderate on the INEF scale; (3) “moderate above-average burnout”(49%)—obtaining moderate results on all three scales (approximately 0.5 SD from the mean) on the EX and CY scales and below 0.5 SD on the INEF); and (4) “very high burnout” (11%)—obtaining high or very high results on the EX and CY scales (above 2SD) and moderate results on the INEF scale (between 0.5 and 1SD from the mean); respondents in this profile had the highest scores on all three scales compared to students in the other groups.

## 5. Discussion

Our goal was to see what burnout profiles can be identified in a group of students in Poland using the LPA method. The hypotheses we put forward, in which we referred to the profiles distinguished by Maslach and Leiter [37], were not confirmed. Hypothesis 1 stated that there are two typical burnout profiles (with three scales with high scores and three with low scores), and hypothesis 2 held the presence of three one-high-dimension profiles. The LPA allowed us to distinguish four student profiles: (1) not burned out at all—this group included moderately effective students, i.e., less effective than might be expected given their effort and dedication; (2) moderately burned-out students, with scores below average; (3) students at risk of burnout—moderately burned out, with scores above average; and (4) severely burned-out students. Apart from the two standard profiles also distinguished by other authors using LPA to analyze burnout [3,31,43] and indicating the presence of a more general burnout phenomenon, our data did not lead us to distinguish any intermediate, only-one-high-dimension profile. Instead, we observed two profiles with two correlated dimensions or two-high-dimensions profiles. These were the following profiles: (1) with two low scores on the exhaustion and cynicism scales and a moderate score on the inefficacy scale and (4) with two high scores on the exhaustion and cynicism scales and a moderate score on the inefficacy scale. We chose not to label the latter profile in such a way as to suggest a one-high-dimension profile, although the score on the cynicism scale was very high, i.e., above 2.5 of the standard deviation from the mean (the difference between EX and CY scores of approximately 0.5 SD); instead, we used the term high level of burnout for this profile because subjects in this group obtained the highest results in all dimensions of burnout compared to the students in the other profiles.

The largest group, almost half of those surveyed, consisted of students who scored moderately—slightly above average. We consider this group at risk of burnout because we recognized that in certain conditions, they might develop burnout. It may seem disturbing that as many as 11% of the subjects had high scores suggesting severe burnout. However, it should also be noted that 40% of the respondents obtained scores indicating at most the moderate level of burnout, although the group of the least-burned-out students was characterized by a sense of moderate efficacy. The largest number of subjects can be included in one of the two profiles in which students scored consistently across the three MBI scales (2 and 3). This could suggest that burnout represents a compact, one-dimensional construct. Then, we could even reduce burnout to chronic fatigue. However, this would be too hasty a conclusion. We cannot ignore other aspects related to the experience of burnout. Our distinction of the severely burned-out student profile “very high burnout” (4) demonstrates that burnout also pertains to students who have become uninvolved and who no longer identify themselves with their studies. Burnout is, therefore, a phenomenon that goes beyond chronic exhaustion with overwhelming responsibilities. Nevertheless, a high score on the cynicism scale was accompanied by a high score on the exhaustion scale.

An interesting role is played by the dimension of inefficacy, for which the results in all profiles were at an average level. It is possible that students experiencing a lack of expected achievement gradually experience burnout. However, based on the results of our study, this could not be determined (as it was cross-sectional). This is especially important because this dimension has often been neglected in the studies on burnout [30].

The resulting four-profile solution identified in the present study partially agrees with a prior study on Italian students [3], in which the authors identified three profiles: burned out, engaged, and overextended students. In our study, we observed neither high nor low INEF scores among the profiles, as if the university environment did not provide recognition for a job well done but also did not create opportunities to develop a sense of efficacy. This indicates a different role of INEF than that of the other two dimensions of burnout. In cases of severe burnout, respondents scored highest on the CY scale in the profile, which may indicate the role of the cultural context in experiencing burnout.

In our study, as in studies by other authors [3,31,43,44], we distinguished two standard profiles with synchronized burnout symptoms, with other researchers distinguishing profiles with high/low scores in all dimensions of burnout, while in our case, these profiles were characterized by moderate intensity on these scales. We also distinguished profiles that were characterized by de-synchrony, with two high or low scores on the scales of exhaustion and cynicism, similar to the desynchronous profiles identified by Lee et al. [44] or Zhang et al. [43]. In contrast, it was not possible to identify desynchronous profiles with only one high score, as in the study by Salmela-Aro and Read [31], who distinguished a profile of ineffective students, or in the study by Portoghese et al. [3], who distinguished an overextended profile with high scores on the exhaustion scale and moderate scores in other dimensions.

Although one can see a growing interest in the search for burnout patterns or typologies of burned-out people, including the use of LPA to conduct such analyses, there is still a small number of studies on burnout profiles in students. In addition, comparing the profiles we obtained with the results of other authors is difficult for at least two reasons. First, researchers use different tools to measure burnout, which may include other dimensions of the phenomenon, and second, they may have a different approach to the relationship between burnout and engagement than the one we adopted in this work; i.e., they may treat burnout and engagement as different phenomena rather than opposite ends of the same continuum.

## 6. Study Limitations and Future Directions

A certain limitation to our study stems from the fact that it was conducted among Polish university students, which makes the obtained results difficult to generalize. A limitation of the sample selection, on the other hand, results from its concentration on students of educational and social sciences and the significant predominance of women in the surveyed group of people, which makes the obtained results difficult to generalize. For future research, it would be interesting to conduct not only a comparative study in an international context (as we only have results for Italian and Estonian students) but also to observe which burnout patterns occur in employees, including representatives of different professions.

Additionally, we consider the transversal nature of our study as its main limitation; in future research, it would be worthwhile to carry out a longitudinal study to answer the question of whether burnout develops due to a lack of achievement that corresponds to the effort invested in work/learning and the level of commitment and identification with the organization. This would also allow us to determine the stability of burnout experienced and to examine what may be most crucial in developing prevention and support processes.

## 7. Conclusions

In conclusion, using a person-centered approach, i.e., LPA, to analyze burnout allowed us to observe that students experience burnout differently. Although two key dimensions (EX and CY) behave similarly, burnout cannot be reduced to the experience of exhaustion alone. Researchers addressing the phenomenon should pay more attention to the dimension of decreased efficacy.

The research results presented in this article provide a voice in the discussion of burnout as a multidimensional construct. The lack of consistency between the results of our study and the results presented by other authors using LPA to analyze burnout requires further exploration in future research.

Leiter and Maslach [37,46] suggested that one-high-dimensional profiles (exhaustion-only and cynicism-only profiles) may provide an early warning sign of developing burnout. The two-dimensional profiles we distinguished (with the cynicism-only profile not too clearly marked, to speak explicitly of a one-high-dimension profile) may indicate a more comprehensive account of the phenomenon in Polish students. This also suggests that exhaustion and cynicism are key dimensions of burnout.

On the other hand, inefficacy, which persisted at a moderate level in each of the highlighted profiles, requires more attention in future research.

## Figures and Tables

**Table 1:** Fit indices for latent profile analysis.

Profiles	LogLik	AIC	BIC	SABIC	Entropy
3	**−5776**	11,609	11,764	11,671	**0.7629**
4	−5735	11,548	**11,756**	11,632	0.7088
5	−5713	**11,523**	11,784	**11,629**	0.6632

**Table 2:** Four-profile LPA model results (means and standard deviations for variables across all profiles).

	Profile 1 (n = 268)	Profile 2 (n = 338)	Profile 3 (n = 741)	Profile 4 (n = 172)	Total (n = 1519)
Exhaustion	2.14 (0.45)	3.03 (0.58)	4.04 (0.78)	5.42 (0.36)	3.63 (1.16)
Cynicism	1.63 (0.31)	2.37 (0.41)	3.71 (0.78)	5.43 (0.31)	3.24 (1.28)
Inefficacy	2.59 (0.87)	2.42 (0.31)	3.45 (0.79)	4.14 (0.82)	3.16 (0.95)

## Data Availability

The database was archived in the Open Science Framework (OSF) repository: (https://osf.io/j9akt/?view_only=eae78c6668d64bd1935d9cc3cec36160) (accessed on 8 March 2023).

## References

[1] Stallman H.M. (2010). Psychological distress in university students: A comparison with general population data. Aust. Psychol..

[2] Schaufeli W.B., Martinez I.M., Pinto A.M., Salanova M., Bakker A.B. (2002). Burnout and Engagementin University Students: A Cross-National Study. J. Cross-Cult. Psychol..

[3] Portoghese I., Leiter M.P., Maslach C., Galletta M., Porru F., D’Aloja E., Finco G., Campagna M. (2018). Measuring Burnout Among University Students: Factorial Validity, Invariance, and Latent Profiles of the Italian Version of the Maslach Burnout Inventory Student Survey (MBI-SS). Front. Psychol..

[4] Niedobylski S., Michta K., Wachoł K., Niedziałek K., Łopuszańska U., Samardakiewicz M., Próchnicki M. (2022). Academic burnout, self-esteem, coping with stress and gratitude among Polish medical students—A cross sectional study. Curr. Probl. Psychiatry.

[5] UNESCO Institute of Statistics Higher Education Gures at a Glance. https://uis.unesco.org/sites/default/files/documents/f_unesco1015_brochure_web_en.pdf.

[6] Cushman S., West R. (2006). Precursors to college student burnout: Developing a typology of understanding. Qual. Res. Rep..

[7] Robotham D. (2008). Stress among higher education students: Towards a research agenda. High. Educ..

[8] Dyrbye L., Thomas M., Power D., Durning S., Moutier C., Massie F.S., Harper W., Eacker A., Szydlo D.W., Sloan J. (2010). Burnout and Serious Thoughts of Dropping Out of Medical School: A Multi-Institutional Study. Acad. Med..

[9] Marôco J., Assunção H., Harju-Luukkainen H., Lin S.W., Sit P.S., Cheung K.-C., Maloa B., Ilic I.S., Smith T.J., Campos J.A.D.B. (2020). Predictors of academic efficacy and dropout intention in university students: Can engagement suppress burnout?. PLoS ONE.

[10] Abreu Alves S., Sinval J., Lucas Neto L., Marôco J., Gonçalves Ferreira A., Oliveira P. (2022). Burnout and dropout intention in medical students: The protective role of academic engagement. BMC Med. Educ..

[11] Lin S.H., Huang Y.C. (2014). Life stress and academic burnout. Act. Learn. High. Educ..

[12] Koeske G.F., Koeske R.D. (1991). Student “Burnout” as a Mediator of the Stress-Outcome Relationship. Res. High. Educ..

[13] Dyrbye L., Thomas M., Massie S., Power D., Eacker A., Harper W., Durning S., Moutier C., Szydlo D.W., Novotny P.J. (2008). Burnout and Suicidal Ideation among U.S. Medical Students. Ann. Intern. Med..

[14] Dyrbye L.N., Shanafelt T. (2016). A narrative review on burnout experienced by medical students and residents. Med. Educ..

[15] Assunção H., Marôco J. (2020). Use of medication in university students with burnout. Psicol. Saúde Doenças.

[16] Salgado S., Au-Yong-Oliveira M. (2021). Student Burnout: A Case Study about a Portuguese Public University. Educ. Sci..

[17] Maslach C., Schaufeli W.B., Leiter M.P. (2001). Job burnout. Annu. Rev. Psychol..

[18] International Classification of Diseases Eleventh Revision (ICD-11, QD85 Burnout), World Health Organization, Geneva License: CC BY-ND 3.0 IGO. https://icd.who.int/en.

[19] Schaufeli W.B., Salanova M., González-Romá A.B., Bakker A. (2002). The Measurement of Engagement and Burnout: A Two Sample Confirmatory Factor Analytic Approach. J. Happiness Stud..

[20] Reisberg L. (2000). Student stress is rising, especially among young women. Chron. High. Educ..

[21] Pieniawska K., Śmiech K., Bar K., Pawlas K. (2017). Can burnout manifest itself in college? A study of Polish medical students’ population. Med. Sr.-Environ. Med..

[22] Walburg V. (2014). Burnout among high school students: A literature review. Child. Youth Serv. Rev..

[23] Maslach C., Schaufeli W.B., Maslach C., Marek T. (1993). Burnout: A multidimensional perspective. Professional Burnout: Recent Developments in Theory and Research.

[24] Maslach C.A., Cooper C.L. (1998). Multidimensional Theory of Burnout. Theories of Organizational Stress.

[25] Demerouti E., Verbeke W.J., Bakker A.B. (2005). Exploring the relationship between a multidimensional and multifaceted burnout concept and self-rated performance. J. Manag..

[26] Olwage D., Mostert K. (2014). Predictors of student burnout and engagement among university students. J. Psychol. Afr..

[27] Robins T.G., Roberts R.M., Sarris A. (2017). The role of student burnout in predicting future burnout: Exploring the transition from university to the workplace. High. Educ. Res. Dev..

[28] Mostert K., Pienaar J. (2020). The moderating effect of social support on the relationship between burnout, intention to drop out, and satisfaction with studies of first-year university students. J. Psychol. Afr..

[29] Jagodics B., Szabó É. (2022). Student Burnout in Higher Education: A Demand-Resource Model Approach. Trends in Psychol..

[30] Mäkikangas A., Kinnunen U. (2016). The person-oriented approach to burnout: A systematic review. Burn. Res..

[31] Salmela-Aro K., Read S. (2017). Study engagement and burnout profiles among Finnish higher education students. Burn. Res..

[32] Freire C., Ferradás M.D.M., Regueiro B., Rodríguez S., Valle A., Núñez J.C. (2020). Coping strategies and self-efficacy in university students: A person-centered approach. Front. Psychol..

[33] Tikkanen L., Pyhältö K., Bujacz A., Nieminen J. (2021). Study Engagement and Burnout of the PhD Candidates in Medicine: A Person-Centered Approach. Front. Psychol..

[34] Vinter K. (2021). Examining academic burnout: Profiles and coping patterns among Estonian middle school students. Educ. Stud..

[35] Meyer J.P., Stanley L.J., Vandenberg R.J. (2013). A person-centered approach to the study of commitment. Hum. Resour. Manag. Rev..

[36] Meyer J.P., Morin A.J.S. (2016). A person-centered approach to commitment research: Theory, research, and methodology. J. Organ. Behav..

[37] Leiter M.P., Maslach C. (2016). Latent burnout profiles: A new approach to understanding the burnout experience. Burn. Res..

[38] Schaufeli W.B., Salanova M. (2007). Efficacy or inefficacy, that’s the question: Burnout and work engagement, and their relationships with efficacy beliefs. Anxiety Stress Coping.

[39] Chirkowska-Smolak T. (2012). Does work engagement burn out? The person-job fit and levels of burnout and engagement in work. Pol. Psychol. Bull..

[40] Maslach C., Jackson S.E., Leiter M.P. (2017). Maslach Burnout Inventory Manual.

[41] Demerouti E., Bakker A.B., Halbesleben J.R.B. (2008). The Oldenburg Burnout Inventory: A Good Alternative to Measure Burnout and Engagement. Handbook of Stress and Burnout in Health Care.

[42] Collins L.M., Lanza S.T. (2010). Latent Class and Latent Transition Analysis for the Social, Behavioral, and Health Sciences.

[43] Zhang X., Klassen R.M., Wang Y. (2013). Academic burnout and motivation of Chinese secondary students. Int. J. Soc. Sci. Humanit..

[44] Lee M.Y., Lee M.K., Lee M.J., Lee S.M. (2020). Academic Burnout Profiles and Motivation Styles Among Korean High School Students. Jpn. Psychol. Res..

[45] Leiter M.P., Maslach C., Friedman H. (1998). Burnout. Encyclopedia o Mental Health.

[46] Maslach C., Leiter M.P. (2008). Early predictors of job burnout and engagement. J. Appl. Psychol..

[47] Schaufeli W., Salanova M. (2011). Work engagement: On how to better catch a slippery concept. Eur. J. Work. Organ. Psychol..

[48] R Core Team 2021 (2015). R: A Language and Environment for Statistical Computing.

[49] Rosseel Y. (2012). Lavaan: An R Package for Structural Equation Modeling. J. Stat. Softw..

[50] Kline R.B. (2013). Principles and Practice of Structural Equation Modeling.

[51] Ferguson S.L., Moore E.W.G., Hull D.M. (2020). Finding latent groups in observed data: A primer on latent profile analysis in Mplus for applied researchers. Int. J. Behav. Dev..

[52] Nylund K.L., Asparouhov T., Muthén B.O. (2007). Deciding on the number of classes in latent class analysis and growth mixture modeling: A Monte Carlo simulation study. Struct. Equ. Model..

